# Structure-guided protein engineering increases enzymatic activities of the SGNH family esterases

**DOI:** 10.1186/s13068-020-01742-8

**Published:** 2020-06-15

**Authors:** Zhengyang Li, Long Li, Yingyi Huo, Zijun Chen, Yu Zhao, Jing Huang, Shuling Jian, Zhen Rong, Di Wu, Jianhua Gan, Xiaojian Hu, Jixi Li, Xue-Wei Xu

**Affiliations:** 1 State Key Laboratory of Genetic Engineering, School of Life Sciences, MOE Engineering Research Center of Gene Technology, Shanghai Engineering Research Center of Industrial Microorganisms, Fudan University, Shanghai, 200438 China; 2 Key Laboratory of Marine Ecosystem Dynamics, Ministry of Natural Resources, Ministry of Natural Resources & Second Institute of Oceanography, Hangzhou, 310012 China

**Keywords:** Esterase, SGNH superfamily, Swapped structure, Alkaline adaptability

## Abstract

**Background:**

Esterases and lipases hydrolyze short-chain esters and long-chain triglycerides, respectively, and therefore play essential roles in the synthesis and decomposition of ester bonds in the pharmaceutical and food industries. Many SGNH family esterases share high similarity in sequences. However, they have distinct enzymatic activities toward the same substrates. Due to a lack of structural information, the detailed catalytic mechanisms of these esterases remain barely investigated.

**Results:**

In this study, we identified two SGNH family esterases, CrmE10 and AlinE4, from marine bacteria with significantly different preferences for pH, temperature, metal ion, and organic solvent tolerance despite high sequence similarity. The crystal structures of these two esterases, including wild type and mutants, were determined to high resolutions ranging from 1.18 Å to 2.24 Å. Both CrmE10 and AlinE4 were composed of five β-strands and nine α-helices, which formed one compact N-terminal α/β globular domain and one extended C-terminal domain. The aspartic residues (D178 in CrmE10/D162 in AlinE4) destabilized the conformations of the catalytic triad (Ser-Asp-His) in both esterases, and the metal ion Cd^2+^ might reduce enzymatic activity by blocking proton transfer or substrate binding. CrmE10 and AlinE4 showed distinctly different electrostatic surface potentials, despite the similar atomic architectures and a similar swap catalytic mechanism. When five negatively charged residues (Asp or Glu) were mutated to residue Lys, CrmE10 obtained elevated alkaline adaptability and significantly increased the enzymatic activity from 0 to 20% at pH 10.5. Also, CrmE10 mutants exhibited dramatic change for enzymatic properties when compared with the wide-type enzyme.

**Conclusions:**

These findings offer a perspective for understanding the catalytic mechanism of different esterases and might facilitate the industrial biocatalytic applications.

## Background

The SGNH-hydrolase family, a superfamily that includes the bacterial lipolytic enzyme GDSL family [1], consists of enzymes possessing four strictly conserved residues, Ser, Gly, Asn, and His, in four conserved blocks, I, II, III, and V, respectively [2, 3]. Among these four residues, Ser and His serve as catalytic residues, and Ser, Gly, and Asn serve as oxyanion hole residues [2, 3]. The SGNH-hydrolase family esterases have a consensus G-D-S-L sequence motif different from the pentapeptide motif GXSXG in most of the other bacterial esterases, and they do not have the nucleophile elbow and cap-domain [4]. To date, only a few structural studies of bacterial SGNH-hydrolase family esterases have been reported [2, 5–8]. Most of them have one molecule in an asymmetric unit characterized by a three-layered α/β/α-fold with a conserved core structure consisting of five β-strands and at least four α-helices. The SGNH superfamily has conserved homologs ranging from prokaryotes to eukaryotes. Furthermore, due to the flexibility of its catalytic domain [9], the SGNH superfamily has multifunctional properties and broad specificities for substrates including carbohydrate esterase [10–12], lipase [13], protease [14], thioesterase [15, 16], arylesterase [15], lysophospholipase [17], and acyltransferase [13, 14]. The SGNH hydrolases from plants are involved in the regulation of development and morphogenesis [18, 19], defense [20], as well as tolerance to environmental stresses [21]. Besides, some SGNH hydrolases, like PlaA [22, 23] and PLB [24], play significant roles in the therapy of mammal diseases. Many SGNH family esterases share high sequence similarity but have distinctly different enzymatic activities toward the same substrates. Benefiting from the development of high-throughput sequencing technology, numerous SGNH hydrolases are found in the genomes of microorganisms, but the function and catalytic mechanism of microbial SGNH hydrolases remain unclear and need to be further explored.

The enzymes isolated from marine organisms usually have many specific features, including temperature adaption, alkaline adaption, salt tolerance, and metal tolerance, which might offer the potential for industrial application. *Croceicoccus marinus* E4A9^T^ was previously isolated from a deep-sea sediment sample [25], and *Altererythrobacter indicus* DSM 18604^T^ was isolated from mangrove-associated wild rice [26]. Both strains belong to the *Erytrobacteraceae* family. According to in silico analysis of whole-genome sequences [27, 28], two novel SGNH-hydrolase family genes, *crme10* and *aline4*, were screened from *C. marinus* and *A. indicus*, respectively. Here, we report the enzymatic characterizations and crystal structures of CrmE10 and AlinE4. These esterases exhibited similar atomic architectures and a similar swap catalytic mechanism; however, their electrostatic surface potentials and enzymatic properties were distinctly different. Structural comparison and structure-based protein engineering showed that mutants with five catalytic reaction-related residues could significantly change enzymatic features. These findings provide new insights into the catalytic mechanism of SGNH-hydrolase family enzymes and might benefit our understanding of their potential uses as biocatalysts.

## Results

### Identification and sequence analysis of CrmE10 and AlinE4

To compare the enzymatic activities of different esterases, the open reading frames of CrmE10 (Accession number: ARU15426.1) and AlinE4 (Accession number: WP_160739227) were identified from the whole-genomes of strains *C. marinus* E4A9^T^ and *A. indicus* DSM 18604^T^, respectively. CrmE10 and AlinE4 have similar amino acid sequences (59.66% identity, 85% coverage). To reveal the relationship between CrmE10 and AlinE4, we performed a phylogenetic analysis with other known lipolytic enzymes using MEGA software [29]. The results showed that both CrmE10 and AlinE4 belong to the SGNH-hydrolase superfamily and the bacterial lipolytic enzyme GDSL family (Additional file 1: Fig. S1). The presence of strictly conserved Ser (residues Ser29 in CrmE10 and Ser13 in AlinE4), Gly (residues Gly66 in CrmE10 and Gly55 in AlinE4), Asn (residues Asn97 in CrmE10 and Asn81 in AlinE4), and His (residues His181 in CrmE10 and His165 in AlinE4) in blocks I, II, III, and V, respectively, confirmed that these two enzymes were new members of the SGNH-hydrolase family [4] (Additional file 1: Fig. S2).

### Biochemical characterizations of CrmE10 and AlinE4

**Fig. 1:**
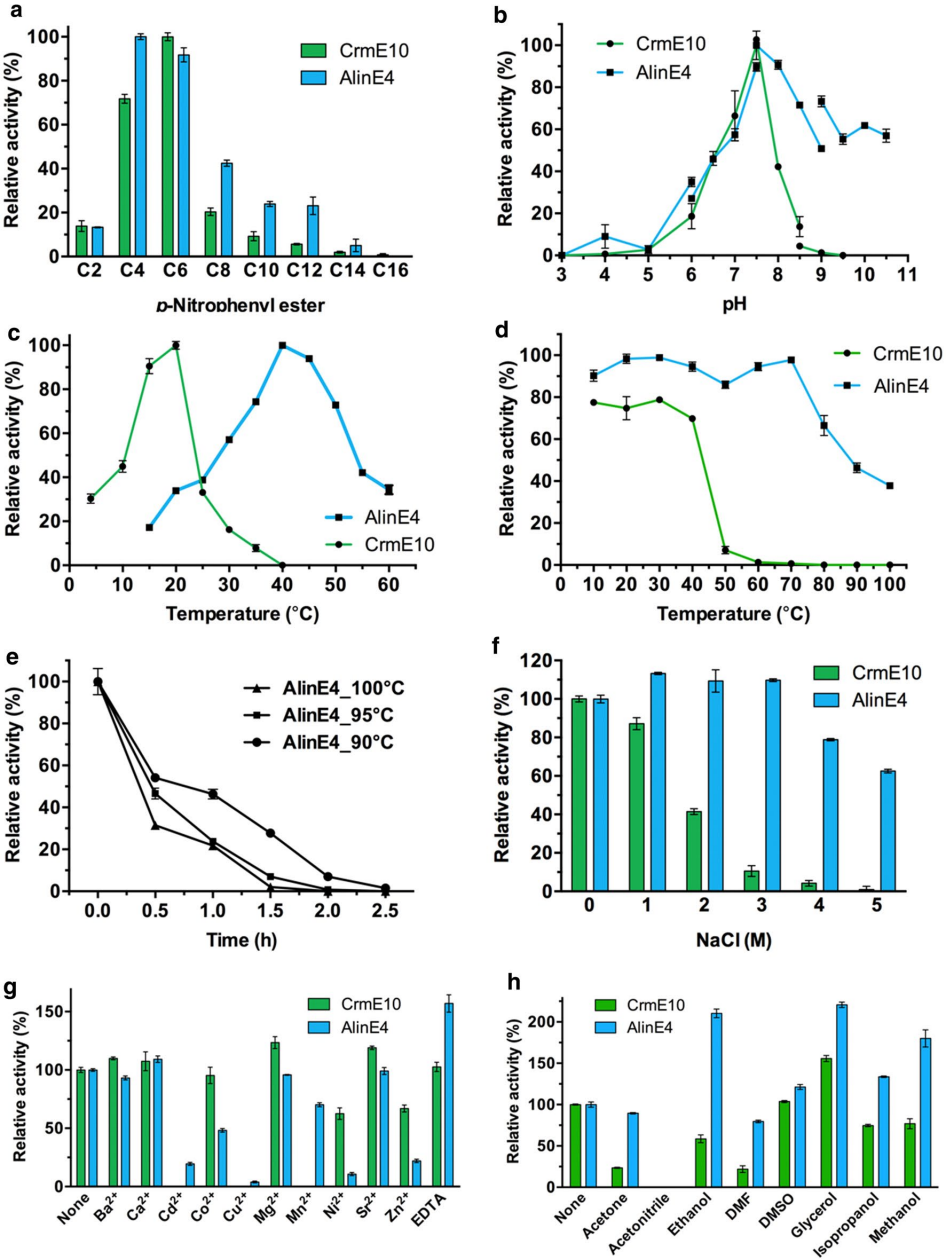
Enzymatic characterizations of CrmE10 and AlinE4. **a** Enzymatic activities toward substrates with various chain lengths of *p*-nitrophenyl (*p*-NP) esters. The value toward *p*-NP hexanoate and *p*-NP butyrate was 100% for CrmE10 and AlinE4, respectively. **b** Effects of different pH on enzyme activities. Enzymatic activities were determined at a series of pH. The value obtained at pH 7.5 was taken as 100%. The gap between different pH due to the buffer changing. **c** Effects of temperature on enzyme activities. Enzymatic activity was determined with a series of temperatures. The values obtained at 20 °C and 40 °C were taken as 100% for CrmE10 and AlinE4, respectively. **d** Effects of temperature on the stability of CrmE10 and AlinE4. The values obtained without heat treatment were taken as 100%. **e** Effects of temperature on AlinE4 enzyme stability after incubation for different times. The values obtained without heat treatment were taken as 100%. **f** Effects of NaCl concentration on the activities. The values obtained without NaCl in the reaction mixture were taken as 100%. **g** Effects of different metal ions on the activities. The values obtained without ions in the reaction mixture were taken as 100%. **h** Effects of organic solvents on the activities. The values obtained without organic solvent were taken as 100%

To better understand the catalytic mechanism for CrmE10 and AlinE4, both enzymes were expressed and purified in *Escherichia coli*. CrmE10 exhibited the highest activity (approximately 29.4 U/mg) toward *p*-nitrophenyl (*p*-NP) hexanoate (C6) at pH 7.5 and 20 °C (*K*_m_, *V*_max_ and *k*_cat_ of 0.16 mM, 33.5 µmol/mg/min and 29.4 s^−1^, respectively) (Fig. 1a–c). AlinE4 displayed the highest activity (approximately 25.8 U/mg) toward *p*-NP butyrate (C4) at pH 7.5 and 40 °C (*K*_m_, *V*_max_ and *k*_cat_ of 0.10 mM, 26.9 µmol/mg/min and 25.8 s^−1^, respectively) (Fig. 1a–c). CrmE10 had about 30% activity at 4 °C and showed no activity above 40 °C, which indicated that CrmE10 was a cold-active enzyme. In contrast, AlinE4 was a mesophilic enzyme with over 32% activity above 60 °C. Interestingly, AlinE4 showed a relatively high thermostability, evidenced by that the enzyme activity was hardly affected after heat treatment at 70 °C for 1 h, and retained 20–40% activity after heat treatment at 100 °C for 1 h (Fig. 1d, e).

CrmE10 showed tolerance to low NaCl concentrations and retained over 83% and 41% of its initial activity at 1 M and 2 M NaCl, respectively (Fig. 1f). The activity of CrmE10 was abolished when NaCl concentrations increased to 5 M. AlinE4 exhibited higher NaCl tolerance, retained over 61% activity at concentrations up to 5 M (Fig. 1f). Metal ions Ba^2+^, Ca^2+^, Mg^2+^, and Sr^2+^ had minimal effects on the activities of the two enzymes (Fig. 1g). Cd^2+^, Cu^2+^, and Mn^2+^ completely abolished the enzymatic activity of CrmE10, but did not work for AlinE4 as these metal ions retained over 20%, 5%, and 70% activities, respectively (Fig. 1g). The addition of Co^2+^, Ni^2+^, and Zn^2+^ decreased the enzymatic activity of CrmE10 over 10%, 35%, and 30%, as well as for the activity of AlinE4 over 51%, 85%, and 79%, respectively (Fig. 1g). The chelating agent EDTA did not decrease the activity of CrmE10 and AlinE4, which indicated that CrmE10 and AlinE4 were not metalloenzyme (Fig. 1g). Besides, compared with CrmE10, AlinE4 showed higher tolerance toward organic solvents, including acetone, ethanol, DMF, DMSO, glycerol, isopropanol, and methanol (15%, v/v) (Fig. 1h).

### Overall structures of CrmE10 and AlinE4

CrmE10 and AlinE4 were expressed in *E. coli* BL21 (DE3) cells as described previously [30]. They were further purified by metal ion affinity chromatography and gel-filtration, finally came out at peak positions of 15 ml (CrmE10) on a Superdex 200 10/300 column and 74 ml (AlinE4) on a Superdex 75 16/600 column, which corresponded to masses of around 22 kDa and 23 kDa, respectively (Additional file 1: Fig. S3A and B). The theoretical molecular weights of CrmE10 and AlinE4 are 22.36 kDa and 20.59 kDa, respectively. The multi-angle light scattering (MALS) results indicated that these two enzymes were monomeric in solution (Additional file 1: Fig. S3C and D).

**Fig. 2:**
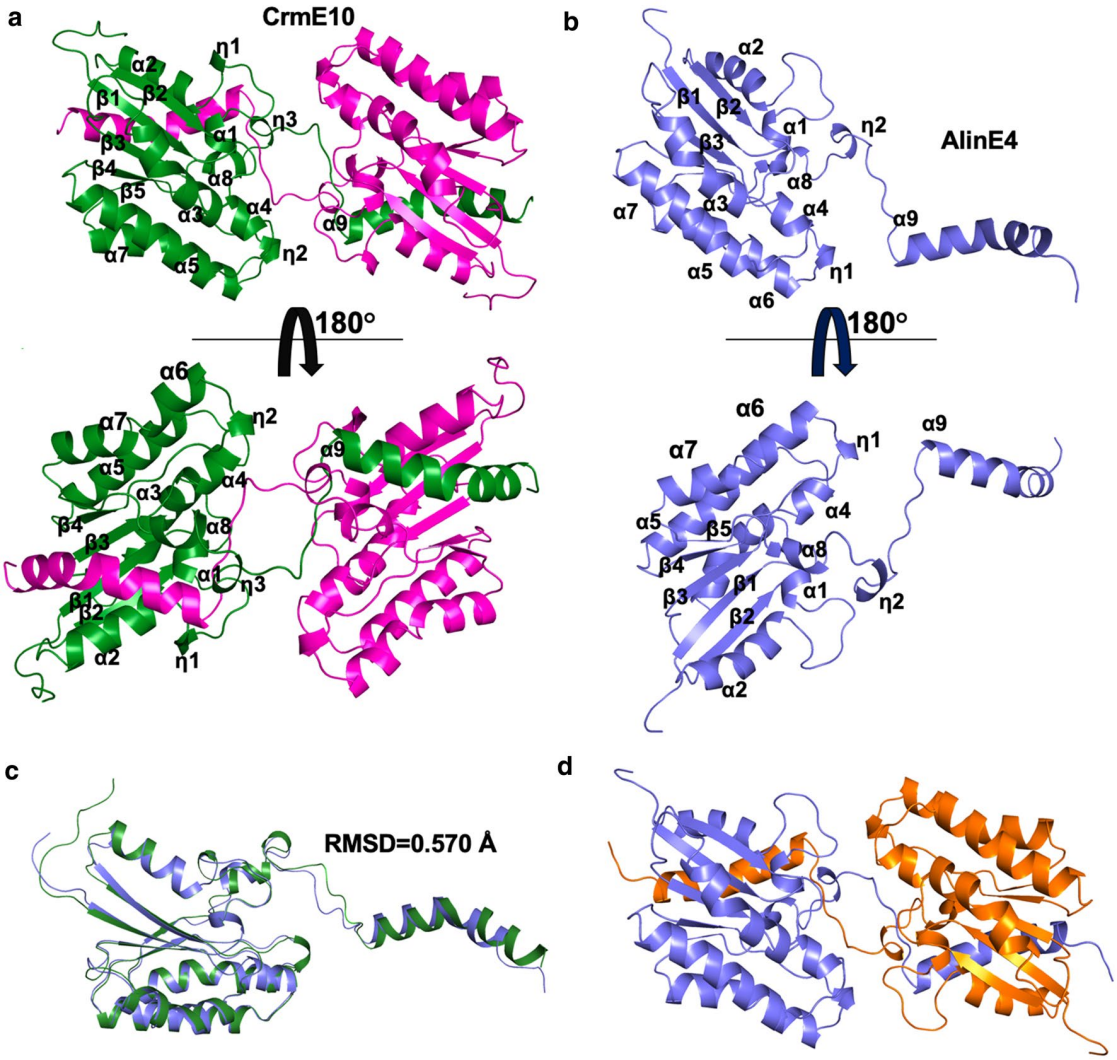
Schematic representations of CrmE10 (PDB: 7C23) and AlinE4 (PDB: 7C82) crystal structures. Cartoon representations of CrmE10 (**a**) and AlinE4 (**b**). The two chains of CrmE10 are labeled in green and magenta; AlinE4 is labeled in blue. **c** The structural superposition of CrmE10 (green) and AlinE4 (blue). The overall structures are very similar with an RMSD value of 0.57 Å. **d** The dimeric model of AlinE4. The two subunits are in blue and orange, respectively

To reveal the molecular basis of CrmE10 and AlinE4, we solved the crystal structures of the two proteins with high resolutions of 1.90 Å for CrmE10 and 1.18 Å for AlinE4, respectively (Additional file 1: Table S1). The CrmE10 structure was determined using the molecular replacement method with esterase TesA (PDB code: 4jgg) as an initial model. In the crystal structure of CrmE10, two identical molecules were found in one asymmetric unit. The CrmE10 structure was composed of 12 α-helices (including three 3_10-_helices) and 5 β-strands. The N-terminal region had a compact architecture that was mainly composed of five predominant β-strands (β1–β5) surrounded by eight α-helices (α1–α8). The C-terminal region was a long α-helix, α9 (Ala184–Asp203), that extended away from the subunit core and wrapped in the other chain. Thus, CrmE10 formed an intertwined dimer though the swapped C-terminal domain (Fig. 2a).

AlinE4 structure was determined using esterase TesA (PDB code: 4jgg) as an initial search model, which revealed similar topology with CrmE10 (Fig. 2b). AlinE4 had one molecule in an asymmetric unit and was consisted of 11 α-helices (including two 3_10-_helices) and 5 predominant β-strands. AlinE4 had a similar topology with CrmE10, as well as the α9 (Ala168-Ala185) wrapped in other chains and formed a symmetric dimer through the swapped C-terminal domain (Fig. 2c and d). The cap-domains and nucleophilic elbows were extensively important components of most other lipolytic enzyme families; however, they were not presented CrmE10 and AlinE4 [31–33]. Furthermore, the PISA analysis for CrmE10 and AlinE4 on the PDBePISA server showed that the multimeric state was 2 for the two enzymes, and the buried areas between two subunits were 8150 Å^2^ (CrmE10) and 8010 Å^2^ (AlinE4), respectively. This suggested CrmE10 and AlinE4 were a dimer in crystal structure.

### Structural comparison of CrmE10 and AlinE4 with other homologs

**Fig. 3:**
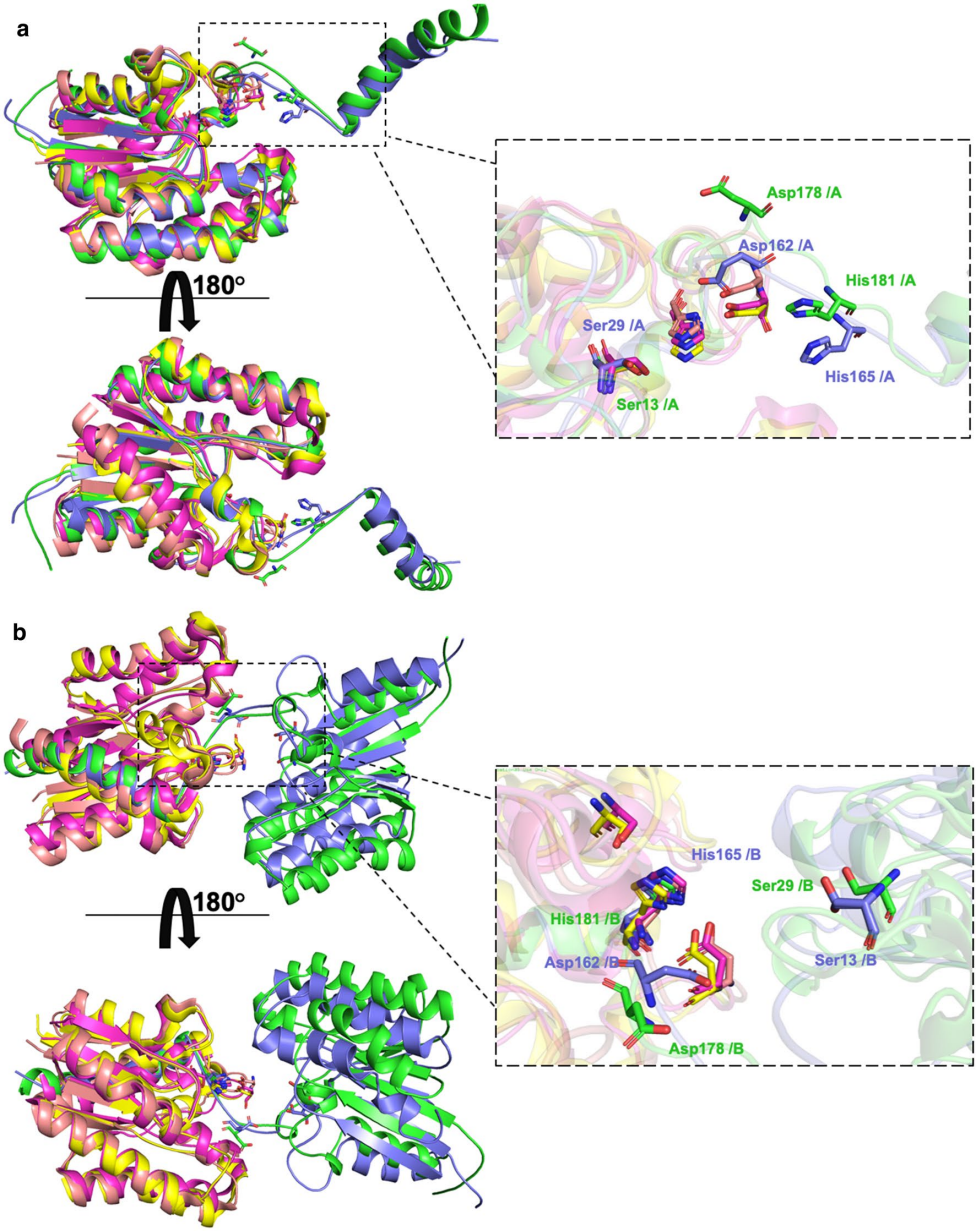
Structure comparison of CrmE10 (PDB: 7C23) and AlinE4 (PDB: 7C82) with other homologs. **a** The structural superposition of CrmE10 (green), AlinE4 (blue), EstA (magenta, PDB code: 3HP4, from *Pseudoalteromonas* sp.), TAP (yellow, PDB code: 1IVN, from *E. coli*), and TesA (orange, PDB code: 4JGG, from *P. aeruginosa*). The major difference was in the catalytic pocket. **b** The structural superposition of the swap chain of CrmE10 and AlinE4 with these homologs. The catalytic residues are indicated with sticks

CrmE10 shared 28.42% (89% coverage), 32.04% (88% coverage), and 34.76% (80% coverage) sequence identities with its homologous EstA (PDB code: 3HP4) [5], TAP (PDB code: 1IVN) [14], and TesA (PDB code: 4JGG) [17], respectively. AlinE4 shared 31.33% (87% coverage), 39.51% (89% coverage), and 39.26% (85% coverage) identities with these proteins, respectively. However, the overall structures among CrmE10, AlinE4, EstA, TAP, and TesA were very similar, evidenced by the low RMSD values of Cα atoms. For CrmE10 with EstA, TAP, and TesA, the values were 1.22 Å, 1.02 Å, and 1.25 Å, respectively. For AlinE4 with EstA, TAP, and TesA, the values were 1.136 Å, 1.082 Å, and 0.979 Å, respectively. The obvious difference was at the loop between α8 and α9 and an α-helix (α9) in the C-terminal region (Fig. 3a). In CrmE10 and AlinE4, the loop and the α9-helix extended away and were wrapped with the other chain in the dimeric structure; whereas in EstA, TAP, and TesA, which have been proved that they can catalyze substrates by one molecule, this region was embedded inward and surrounded by predominant β-strands with other helices. However, in CrmE10 and AlinE4, the α8–α9 loop and α9-helix of the other chain were highly conserved with the same regions, suggesting that the catalytic reaction of CrmE10 and AlinE4 required coordination of two molecules, which might be a new mechanism of SGNH-hydrolase family esterases (Fig. 3b).

### Dimerization contributed to the catalytic activities of CrmE10 and AlinE4

**Fig. 4:**
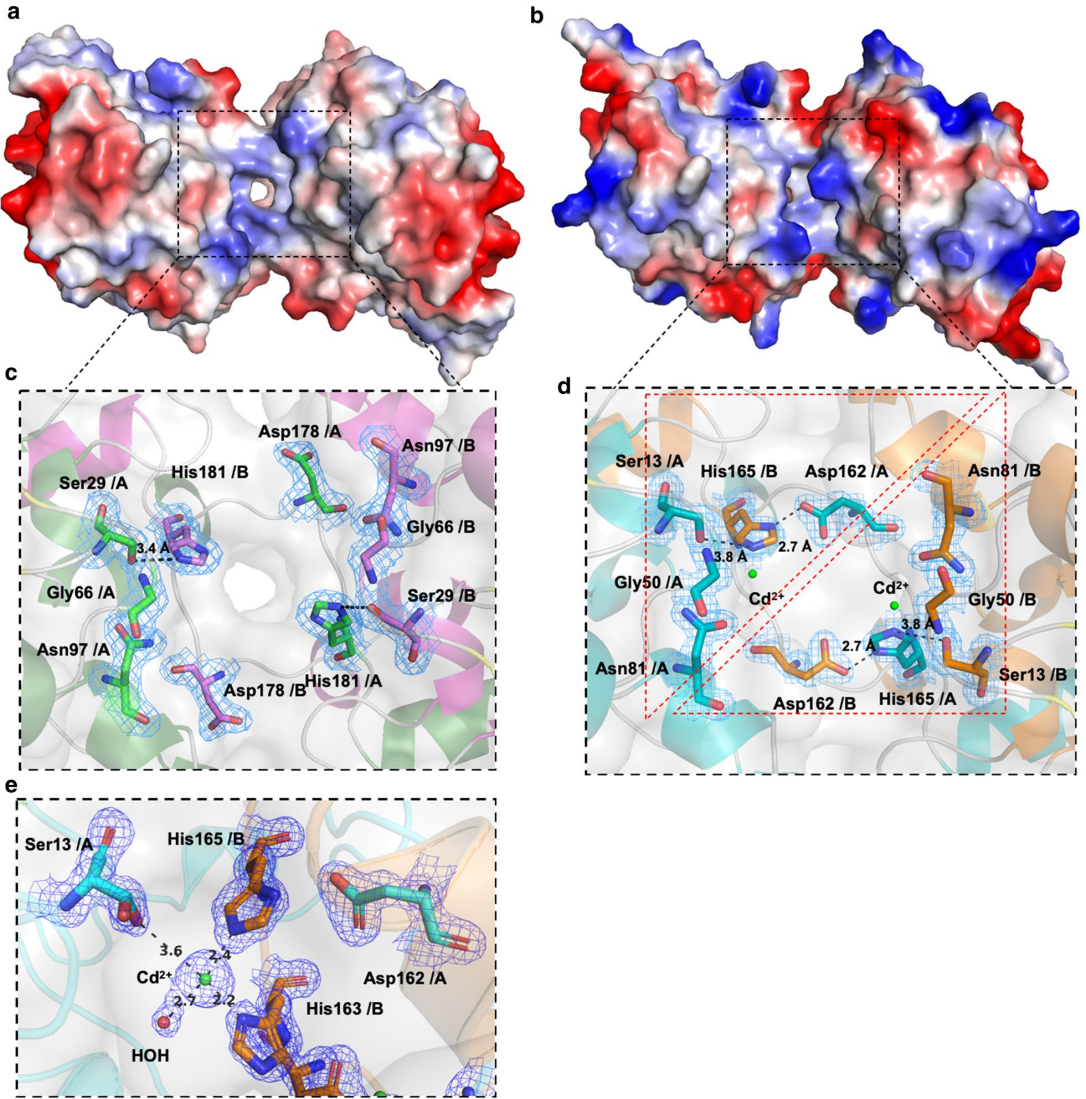
Visualization of the catalytic sites of CrmE10 (PDB: 7C23) and AlinE4 (PDB: 7C82). CrmE10 (**a**) and AlinE4 (**b**) are shown with electrostatic potential surfaces. Red: negative potential; blue: positive potential. The residues of the catalytic triad and oxyanion hole of CrmE10 (**c**) and AlinE4 (**d**) are shown as stick models. The Cd^2+^ ions in AlinE4 are shown as green spheres. The electronic density map is contoured to 1.0 σ at the 2*Fo*-*Fc* map. The dashed lines denoted hydrogen bonds. **e** The metal ion Cd^2+^ interacted with residues in the catalytic triad. The corresponding residues are indicated with a stick model. The electronic density map is contoured to 1.0 σ at the 2*Fo*-*Fc* map. The Cd^2+^ is shown as a green ball and the water molecule is shown as a red ball

According to sequence alignment, the catalytic triad of CrmE10 consisted of Ser29, Asp178, and His181, and it was composed of Ser13, Asp162, and His165 in AlnE4 (Additional file 1: Fig. S2). Mutants CrmE10-S29A, CrmE10-D178A, and CrmE10-H181A had no enzymatic activities, which confirmed that these residues were crucial for the activity (Additional file 1: Fig. S4A). In CrmE10 and AlinE4 structures, catalytic residue Ser was located on helix α1, and residues Asp and His were located on the loop between helix η3 (CrmE10) or helix η2 (AlinE4) and helix α9, which were typically different from other esterases. Interestingly, the catalytic triads of CrmE10 and AlinE4 were not composed of Ser, Asp, and His from the same chain, which was common in other esterases (Fig. 4). For AlinE4, residues Ser13 and Asp162 on chain A and His165 on chain B were in a reasonable position of the catalytic triad, of which hydrogen bonds within the catalytic triad could be formed from Ser13-Oγ to His165-Nε2 and from His165-Nδ1 to Asp162-Oδ1 (Fig. 4b, d). In mutants AlinE4-S13A, AlinE4-D162A, and AlinE4-H165A, catalytic activities toward *p*-NP esters were almost abolished (Additional file 1: Fig. S4B). Moreover, substrates *p*-NP butyrate and *p*-NP hexanoate could be successfully docked into the active sites of AlinE4 and CrmE10 using AutoDock software, respectively (Additional file 1: Fig. S4C and D). In CrmE10, the active sites Ser and His also formed hydrogen bonds between Ser29-Oγ and His181-Nε2 (3.4 Å/3.9 Å) (Fig. 4a, c).

As the catalytic triad was composed of the swapped dimeric structure, to further identify dimerization contributes to the catalytic activities of CrmE10 and AlinE4, mutagenesis analysis was performed for residues Asp178 and Ser29. The polycistronic plasmid CrmE10-W1 was composed of His-sumo-tagged WT CrmE10 and untagged mutant CrmE10-D178A, whereas the plasmid CrmE10-W2 was composed of His-sumo-tagged CrmE10 and untagged mutant CrmE10-D178A-S29A. The heated proteins did not have enzymatic activities and were used as a control. After expression and purification from *E. coli*, CrmE10-W1 had higher enzymatic activity than CrmE10-W2 (Additional file 1: Fig. S4A). As mutants CrmE10-S29A and CrmE10-D178A had no activity, the results showed that residues Ser29 and Asp178 influenced the enzymatic activity of CrmE10 by domain swapping. Thus, dimerization participated in the active sites of CrmE10, and this mechanism might also fit AlinE4 due to the similar architecture, which was different from other homologs.

### Metal ion Cd^2+^ affected the catalytic activity of AlinE4

The mechanism of SGNH-hydrolase family esterase activity involves a two-step reaction (acylation and deacylation) similar to those proposed for lipolytic enzymes and serine proteases [34]. In this reaction, Ser is a nucleophile residue, and His is the proton donor/acceptor [32]. Many heavy metal ions have impacts on enzymatic activity, but the mechanism was not clear [12, 35–37]. There was one density map around the catalytic sites in the crystal structure of AlinE4, which turned out to be one Cd^2+^ evidenced by electrochemistry analysis (Additional file 1: Table S2). According to the characterization of AlinE4, Cd^2+^ had negative impacts on the enzymatic activity of AlinE4, evidenced by only retaining 20% activity at 10 mM CdCl_2_ (Fig. 1g). In the crystal structure of AlinE4, Cd^2+^ interacted with residues Ser13 and His165, which were components of the catalytic triad (Fig. 4e). The results suggested that Cd^2+^ might act on activity through (i) blocking proton transfer and (ii) protecting substrates from nucleophile attack.

### Structure-based mutation dramatically increased the enzymatic activity

CrmE10 and AlinE4 shared similar atomic architectures (RMSD value of Cα was 0.570 Å, Fig. 2c); however, the enzymatic properties exhibited significant difference, including substrate specificity, alkaline adaptability, temperature adaptability, metal ion tolerance, and organic solvent tolerance (Fig. 1a–h). To further investigate the possible mechanism, we analyzed the sequences based on the 3D structures and found five specific residues might contribute to these enzymatic property difference. The residues were acidic in CrmE10, including Asp77, Glu86, Asp123, Glu159, and Asp200, whereas the corresponding residues were basic in AlinE4, including Lys61, Lys70, Lys107, Lys143, and Lys184 (Additional file 1: Fig. S2). The region of these five sites in CrmE10 formed a negative potential surface; however, the corresponding region was full of positive potential in AlinE4.

**Fig. 5:**
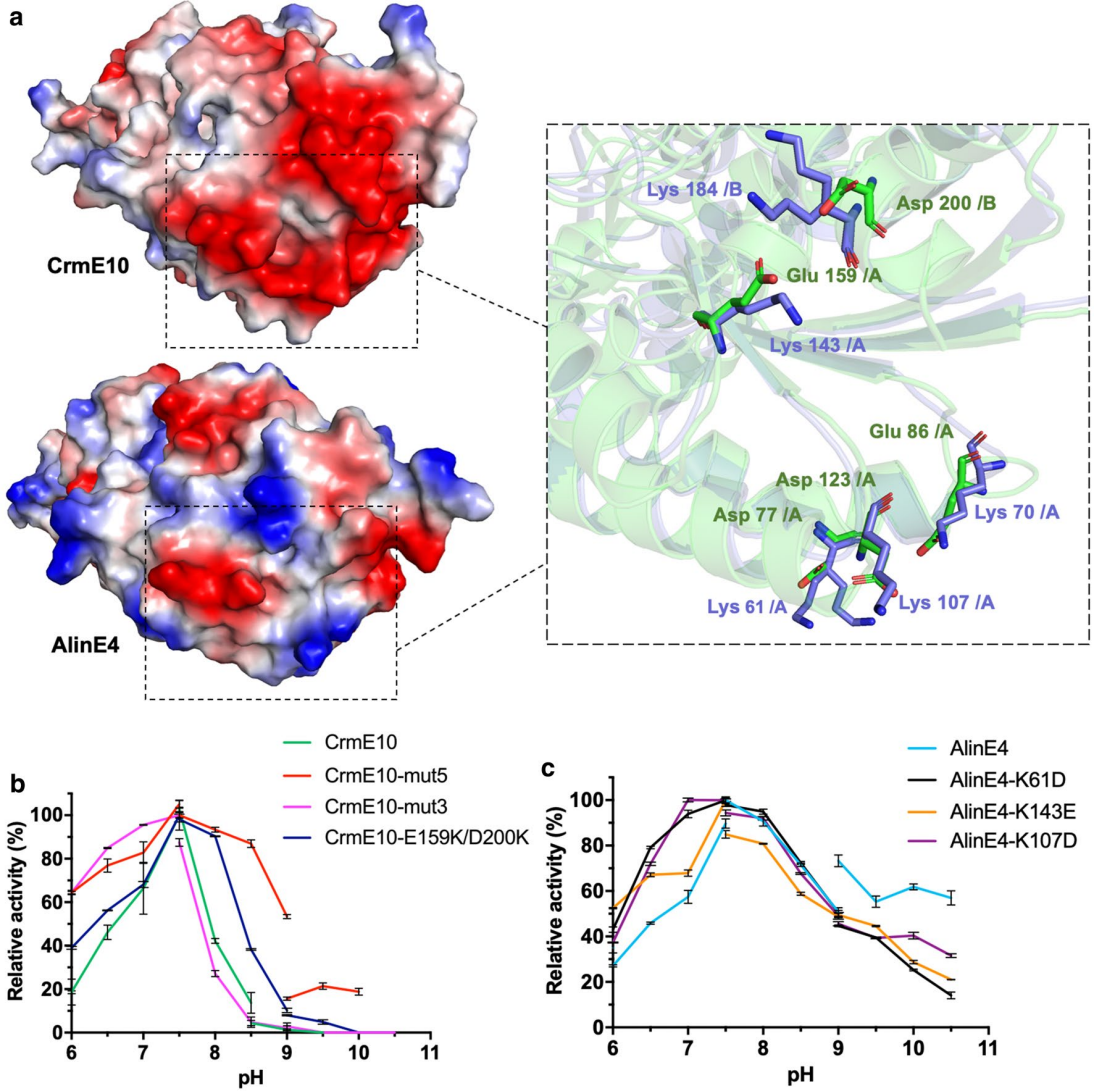
The key residues associated with alkaline adaptability. **a** CrmE10 (top, PDB: 7C23) and AlinE4 (bottom, PDB: 7C82) are indicated in electrostatic potential surfaces. Red: negative potential; blue: positive potential. Five residues associated with of alkaline adaptability are indicated in sticks (green: CrmE10, blue: AlinE4). Enzymatic activities of CrmE10 (**b**) and AlinE4 (**c**) and their mutants determined with a range of different pH values. The value obtained at pH 7.5 was taken as 100%. The gap was between different pH due to the buffer changing

AlinE4 exhibited high alkaline adaptability evidenced by retaining about 60% activity at pH 10.5, whereas CrmE10 only retained about 10% activity at pH 8.5 (Fig. 1b). The enzymatic assay results showed that mutants AlinE4-K61D, AlinE4-K107D, and AlinE4-K143E retained about 45% or lower activity when pH was equal to or higher than 9.0 (Fig. 5c). The engineered enzyme CrmE10-mut5 (D77K/E86K/D123K/E159K/D200K) significantly increased to about 20% activity at pH 10, whereas CrmE10-E159K/D200K increased a little enzymatic activity at pH 9.0–9.5 and CrmE10-mut3 (D77K/E86K/D123K) had no difference as compared to wild-type CrmE10 (Fig. 5b), which meant these five sites synergistically participated in alkaline adaptability. The mutants of these five residues might change the surface charge of CrmE10 and increase its stability in an alkaline environment, therefore increasing its activity. AlinE4 had higher enzymatic activity toward long-chain substrates (C8, C10, C12 and C14) than CrmE10 (Fig. 1a). Compared with wild-type CrmE10, CrmE10-mut5 exhibited higher activity toward long-chain substrates (Additional file 1: Fig. S5A), which suggested these five sites might contribute to substrate binding. Furthermore, the temperature preferences of mutants CrmE10-mut5 and CrmE10-E159K/D200K were different from wild-type CrmE10 (Additional file 1: Fig. S5B). Mutants CrmE10-mut5 and CrmE10-E159K/D200K exhibited more than 50% activity with the addition of 10 mM Cd^2+^ or Mn^2+^, which completely inhibited wild-type CrmE10 activity. Moreover, these two engineered enzymes had lower activities with the addition of 10 mM Ni^2+^ than wild-type CrmE10, which was similar to AlinE4 (Additional file 1: Fig. S5C). Besides, compared with wild-type CrmE10, CrmE10-mut5 exhibited higher activity with the addition of DMF or DMSO (Additional file 1: Fig. S5D). Therefore, changing the charge properties of the esterases would dramatically affect the enzymatic properties.

## Discussion

**Fig. 6:**
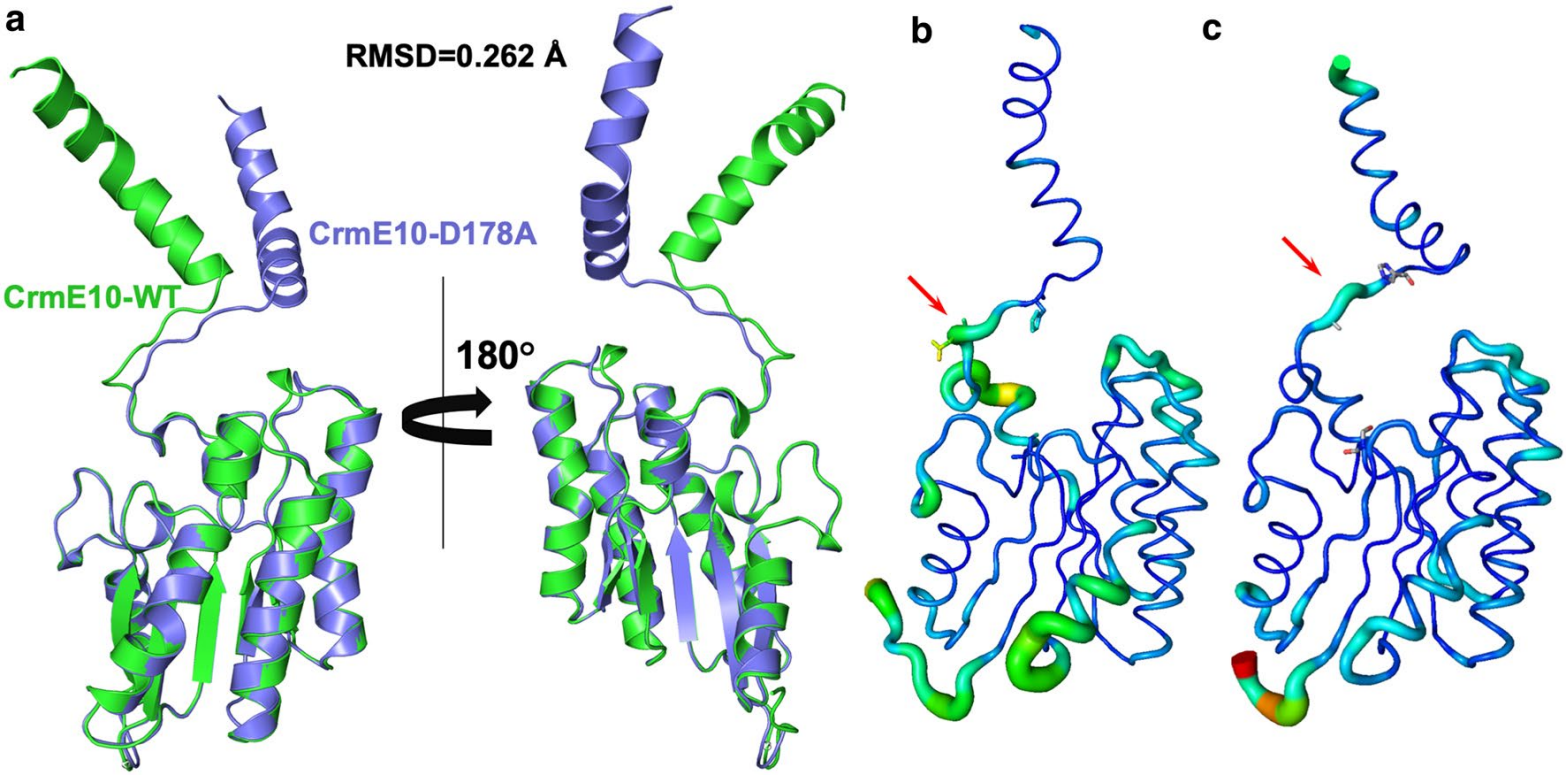
Structural comparison of CrmE10 (PDB: 7C23) and its mutant CrmE10-D178A (PDB: 7C29). **a** The structural superposition of CrmE10 (green) and CrmE10-D178A (blue). **b**, **c** The B-factor distribution of CrmE10 and CrmE10-D178A. Wider and redder tubing corresponds to higher B-factor. Red arrows indicate the flexible loops between 3_10_-helices η3 and α-helix α9 of CrmE10

SGNH-hydrolase family esterases play essential roles in food, pharmaceutical, and biological industries [3]. Our study revealed a new catalytic mechanism for two esterases from different sources. The swapped domains in the dimers of CrmE10 and AlinE4 contributed to the enzymatic reaction, which was different from the catalytic mechanism in other esterases, such as EstA [5], TesA [17], and TesI [14]. Although the MALS results suggested that CrmE10 and AlinE4 are monomers in solution (Additional file 1: Fig. S3C and D), the crystal structures and PISA analysis showed that the two esterases could form dimers by the swapped domains. The dimeric structures could contribute to the enzymatic activities and help the stabilization for crystal packing (Additional file 1: Fig. S4), which was similar to the reported human archease [38]. The esterases SsEst from *Streptomyces scabies* and NanS from *E. coli* were reported to possess catalytic Ser-His dyads [7, 39]. The correct orientation of the imidazole ring of His in SsEst was ensured by a hydrogen bond between His-Nδ1 and a main chain carbonyl oxygen [7]. In NanS, only the catalytic dyad residues Ser and His were essential for catalysis, and it was hypothesized that the hydroxyl ion played the catalytic role or that substrate binding caused active site reorganization [39]. In SGNH-hydrolase family enzymes, the residue Ser on the catalytic triad was considered an essential element for aromatic acyl substrate binding, and His was considered as the proton donor/acceptor [32, 40]. However, the function of the Asp residue was barely investigated. The loop between 3_10_-helix η3 and helix α9, on which the catalytic Asp residue was located, showed significant conformational change between the WT and mutant CrmE10 (Fig. 6a) and had a higher B-factor (Fig. 6b, c). The higher B-factor of the loop implied flexibility in its structure and function. Hence, this Asp might participate in destabilizing the conformation of the loop in the resting state and in changing it to a more stable conformation when the substrate bound the enzyme. Although the mutant AlinE4-D162A did not show significant variation compared with wild-type AlinE4, the B-factor of this loop was lower than wild-type AlinE4 (Additional file 1: Fig. S6). These results revealed that the Asp in the catalytic triad destabilized the conformation of the reaction pocket.

Currently, many protein engineering methods have been developed to improve esterases (or lipases) properties, including substrate specificity [41, 42], activity [43, 44], thermostability [45], and enantioselectivity [46, 47]. However, limited studies for enhancing alkali tolerance have been reported, especially based on structural information. Although esterase CrmE10 and AlinE4 exhibited similar atomic architectures as well as similar swap catalytic mechanisms, they had distinctly different electrostatic surface potentials, as well as different alkaline adaptability, thermal stability, salt tolerance, and heavy metal ions tolerance (Figs. 1 and 2). Structure-guided mutation in aspartic acid/glutamic acid to lysine increased the alkaline adaptability (Fig. 5). The mutants of CrmE10-mut5 and CrmE10-E159K/D200K exhibited different properties from wild-type CrmE10 (Additional file 1: Fig. S5). According to previous studies, the thermal stability can be affected by (i) more abundant, high hydrophobicity, charged residues (such as Glu, Arg, and Lys) rather than uncharged polar amino acid (such as Ser, Thr, Asn, and Gln) in the amino acid sequences [41, 48, 49]; (ii) more α-helices in structures [50, 51]; and (iii) some intermolecular forces, such as hydrogen bonds [52]. AlinE4 has 6 glutamine residues and 12 lysine residues, while CrmE10 has 12 glutamine residues and only 1 lysine residue, which might lead to the difference in thermal stability. The salt tolerance might be affected by charges on the surface. When the surface charges were calculated using PyMOL software, the values of CrmE10 and AlinE4 were −37 and 0, respectively, which indicated AlinE4 had a nonpolar surface and its enzymatic activity was not susceptible to salt. Therefore, structure-guided protein engineering for hydrolases might increase the potential industrial and pharmaceutical uses through simply changing the charged residues.

## Conclusions

Here, we reported the enzymatic characterizations and crystal structures of marine bacterial esterases CrmE10 and AlinE4. The two enzymes shared high sequence similarity and similar atomic architectures; however, they had significantly different enzymatic activities. Despite sharing a similar swapping catalytic mechanism, CrmE10 and AlinE4 had distinctly different electrostatic surface potentials. Structure-based mutation showed that CrmE10 obtained elevated alkaline adaptability and had a significant increase in enzymatic activity from 0 to 20% at pH 10.5 when five acidic residues were mutated to the corresponding basic residues in AlnE4. In addition, one key residue (Asp162 in AlinE4/Asp178 in CrmE10) was found to be able to stabilize the conformation of both esterases and the metal ion Cd^2+^ reduced enzymatic activity by blocking proton transfer and substrate binding. Our current findings offer a perspective for understanding the catalytic mechanism of esterases and could facilitate industrial biocatalytic applications of these esterases.

## Materials and methods

### Cloning, mutation, protein expression, and purification

*Croceicoccus marinus* E4A9^T^ was previously isolated from a deep-sea sediment sample [25] and was stored in our lab. *Altererythrobacter indicus* DSM 18604^T^ was isolated from mangrove-associated wild rice [26] and was purchased from Leibniz Institute DSMZ-German Collection of Microorganisms and Cell Cultures. The genes *crme10* and *aline4* were cloned into plasmid pSMT3 to produce the N-terminal His-Sumo tagged fusion proteins from the genomic DNA of *C. marinus* E4A9^T^ and *A. indicus* DSM 18604^T^, respectively. Point mutants were generated by site-directed mutagenesis using wild-type plasmids as templates for PCR. After digested with *Dpn*I enzyme, the products were transformed into *E. coli* DH5α cells and determined by DNA sequencing. CrmE10-W1 contains wild-type CrmE10, ribosomal binding site (RBS) and CrmE10-D178A in the pSMT3 plasmid, whereas the polycistronic CrmE10-W2 plasmid contains wild-type CrmE10, ribosome binding sites, and CrmE10-D178A-S29A. The wide-type and mutated proteins were expressed in *E. coli* BL21 (DE3) cells. The cells were cultured in LB broth medium induced by adding 0.5 mM isopropyl-*β*-d-thiogalactoside (IPTG), when OD_600_ came to 0.6–0.8. After cultivation at 16 °C for 20 h, cells were harvested by centrifugation at 6000 rpm for 15 min at 4 °C, resuspended in starting buffer (50 mM Tris–HCl, 500 mM NaCl, 10 mM imidazole, 5% glycerol, pH 8.0), and disrupted by French press homogenizer (JNBio, China). Cell debris was removed by centrifugation and the supernatant was incubated with Ni Sepharose (GE, USA) for 1 h. After washing with buffer 1 (50 mM Tris–HCl, 500 mM NaCl, 50 mM imidazole, 5% glycerol, pH 8.0), the recombinant protein was eluted with buffer 2 (50 mM Tris–HCl, 500 mM NaCl, 250 mM imidazole, 5% glycerol, pH 8.0). Subsequently, the His-Sumo tag was removed by overnight digestion with the ULP1 enzyme. The recombinant target proteins were further purified by gel-filtration using the Superdex 200 10/300 column (GE, USA) in a buffer (20 mM Tris–HCl, 100 mM NaCl, 2 mM DTT, pH 7.4). The fractions of elution were determined by sodium dodecyl sulfate polyacrylamide gel electrophoresis (SDS–PAGE). Furthermore, the protein concentrations were determined by the Bradford method [53] with bovine serum albumin (BSA) as standard.

### Sequence analysis

Multiple alignments of amino acid sequences were performed using ClustalX v.2 program [54]. Secondary structure alignment was generated by DSSP v.2.0 [55] and ESpript v.3.0 (http://espript.ibcp.fr/ESPript/ESPript/) [56]. The phylogenetic tree was constructed by the neighbor-joining method using MEGA (Molecular Evolutionary Genetics Analysis) v.7.0 software [29].

### Multi-angle light scattering (MALS) analysis

MALS analysis was performed in the National Center for Protein Science Shanghai (NCPSS). 20 μl of 1 mg/ml purified target protein was subjected to SEC-MALS using a WTC-030S5 size-exclusion column (Wyatt, USA) with elution buffer (20 mM Tris–HCl, pH 7.4, 100 mM NaCl) and passed in tandem through a Wyatt DAWN HELEOS II light scattering instrument (Wyatt, USA) and an optilab refractometer (Wyatt, USA). Data collection and analysis were performed with Astra 6 software (Wyatt, USA).

### Enzymatic activity assays

Esterase activity assays were performed using a spectrophotometric method with the appropriate amount of purified enzyme in standard reaction buffer containing 100 mM Tris–HCl (pH 7.5), enzyme (concentration at 1–5 μg/mL) and 1 mM *p*-nitrophenyl (*p*-NP) hexanoate (for CrmE10 and its mutants, TCI, Japan), or *p*-NP butyrate (for AlinE4 and its mutants, Sigma-Aldrich, USA). The enzymatic activity was determined at 20 °C (for CrmE10 and its mutants) or 40 °C (for AlinE4 and its mutants) by measuring the amount of releasing *p*-nitrophenol using Beckman Coulter DU 800 UV/Visible spectrophotometer (Beckman, USA) at 405 nm. All values were measured in triplicates and corrected for the autohydrolysis of the substrates. One unit of enzymatic activity was defined as the amount of enzyme required for releasing 1 μmol of *p*-nitrophenol per minute from the *p*-nitrophenyl ester. The kinetic parameters (*K*_m_ and *V*_max_) were calculated from enzymatic activity measurements with *p*-NP hexanoate (for CrmE10) or *p*-NP butyrate (for AlinE4) ranging from 0.05 mM to 2 mM. Initial reaction velocities measured at various concentrations were fitted to the Lineweaver–Burk transformation of the Michaelis–Menten equation [57].

The optimum pH of esterases CrmE10 and AlinE4 was determined over the pH range from 3.0 to 9.5. The buffers included citrate buffer (100 mM, pH 3.0–6.0), phosphate buffer (100 mM, pH 6.0–7.5), Tris–HCl buffer (100 mM, pH 7.5–8.5 or 7.5–9.0), and CHES–NaOH buffer (50 mM, pH 8.5–10.5 or 9.0–10.5). The enzymatic activity was measured under 348 nm. The effects of temperature on esterases CrmE10 and AlinE4 were measured over a range of 15–60 °C. For investigation of the thermostability, the residual activity of CrmE10 and AlinE4 was determined after incubation at various temperatures ranging from 10 °C to 100 °C for 1 h. And the thermostability of AlinE4 was further determined after incubation at 90 °C, 95 °C and 100 °C for 0–2.5 h.

Various chain lengths of *p*-NP esters, including *p*-NP acetate (C2), *p*-NP butyrate (C4), *p*-NP hexanoate (C6) (TCI, Japan), *p*-NP octanoate (C8), *p*-NP decanoate (C10), *p*-NP dodecanoate (C12), myristate (C14), and *p*-NP palmitate (C16) (Sigma-Aldrich, USA, unless otherwise stated) were added into the reaction buffer with final concentration of 1 mM for determining substrate specificity.

The effects of NaCl on CrmE10 and AlinE4 activity were evaluated by adding 0–5 M NaCl to the assay mixture. The effects metal ions were measured using various divalent cations, namely Zn^2+^, Sr^2+^, Ni^2+^, Mn^2+^, Mg^2+^, Co^2+^, Ca^2+^, and Ba^2+^, at final concentration of 10 mM. The effect of the chelating agent ethylenediaminetetraacetic acid (EDTA) was determined at a final concentration of 10 mM. The effects of organic solvents were determined using acetone, acetonitrile, ethanol, dimethylformamide (DMF), dimethyl sulfoxide (DMSO), glycerol, isopropanol, and methanol, at a final concentration of 15% (v/v).

### Electrochemistry analysis

Several crystals of AlinE4 were washed in a Tris buffer without heavy metal ions for several seconds and transferred into a micro-centrifuge tube. Then the crystals were dissolved completely with the Tris buffer. The protein concentration was measured by the Bradford method [53]. The 884 professional VA (Metrohm Co., Ltd) was used to measure the type of ion through the Anodic Stripping Voltammetry [58–60], and the concentration of cadmium was calculated by comparing with the standard solution. All experiments were repeated three twice.

### Crystallization and X-ray data collection

CrmE10 and AlinE4 were applied to crystallization trials carried out at 20 °C by hanging- and sitting-drop vapor-diffusion methods by mixing 30 mg/ml protein with an equal volume reservoir solution. The crystals of native CrmE10 were grown in a reservoir solution containing 150 mM calcium acetate, 100 mM imidazole–HCl (pH 8.0), and 10% PEG 8000. The crystals were briefly soaked in 25% (v/v) glycerol dissolved in their reservoir solution, as a cryoprotectant solution, before being flash-frozen directly in liquid nitrogen. The CrmE10-D178A crystals were grown in the condition of 250 mM calcium acetate, 100 mM imidazole–HCl (pH 8.5), 5% PEG 1000, and 3% 1,6-Hexanediol. The cryoprotectant solution of the CrmE10-D178A crystals was 20% (v/v) PEG 400. The crystals of native AlinE4, AlinE4-S13A, and AlinE4-D178A were grown in 1 M NaAc, 100 mM HEPES, and 50 mM CdSO_4_. All X-ray diffraction datasets were collected at BL17U1 [61], BL18U1, and BL19U1 [62] beamlines of the Shanghai Synchrotron Radiation Facility (SSRF, China). Diffraction data were integrated and scaled using software HKL2000 [63].

### Structure analysis and refinement

The crystal structures of wild-type (WT) CrmE10 and AlinE4 were determined by molecular replacement using esterase TesA (PDB code: 4jgg) [17] as the search model. The mutant protein structures were solved using the WT structure as the search model. After cycles of refinement and model building processed using program REFMAC5 [64, 65] of CCP4i and software COOT [66], the crystallography R-free and R-factor values reached to the satisfied range. PROCHECK [67] of PDBsum was used to evaluate the quality of the final 3D-structures. The other homologous structures were identified using DALI server [68, 69] and blast program (https://blast.ncbi.nlm.nih.gov/Blast.cgi). Substrate docking studies were performed using the AutoDockTools4 program [70]. All the 3D-structures were analyzed and displayed using the PyMOL molecular graphics system (The PyMOL Molecular Graphics System, Version 2.0 Schrödinger, LLC). CrmE10, CrmE10-D178A, AlinE4, AlinE4-D162A, and AlinE4-S13A were deposited to Protein Data Bank with accession codes 7C23, 7C29, 7C82, 7C84, and 7C85, respectively. Data collection and refinement parameters are listed in Additional file 1: Table S1.

## Supplementary information

**Additional file 1.** Additional figures and tables.

## Data Availability

The X-ray datasets generated for this study were deposited in the Protein data bank (PDB, http://www.rcsb.org/). The PDB codes of CrmE10, CrmE10-D178A, AlinE4, AlinE4-D162A, and AlinE4-S13A were 7C23, 7C29, 7C82, 7C84, and 7C85, respectively.

## Abbreviations

*p*-NP: *p*-Nitrophenyl
MALS: Multi-angle light scattering
RMSD: Root-mean-square deviation
DMF: *N,N*-Dimethylformamide
DMSO: Dimethyl sulfoxide
