# Supplementary material for: Structure-guided protein engineering increases enzymatic activities of the SGNH family esterases
Source: Biotechnol Biofuels. 2020 Jun 15;13:107. doi: 10.1186/s13068-020-01742-8 (PMC7294632; doi:10.1186/s13068-020-01742-8)
Supplement: Supplementary file 1 — Additional file 1. Additional figures and tables. [file 13068_2020_1742_MOESM1_ESM.docx]

**Table S1. Statistics for data collection and refinement.**

1. CrmE10

| Items | CrmE10 | CrmE10-D178A |
| --- | --- | --- |
| **Data collection** |  |  |
| Wavelength | 0.97923 | 0.9778 |
| Resolutions (Å) | 50.00-1.9 (1.96-1.9)^a^ | 50.00-2.18 (2.22-2.18) |
| Space group | C 2 | P 2_1_ 2_1_ 2 |
| Unit cell (Å, °) | a=116.655, b=37.019, c=99.747  α=90, β=99.055, γ=90 | a=65.012, b=70.041, c=80.572  α=β=γ=90 |
| Unique reflections | 33196 (3319) | 19765 (1927) |
| Completeness (%) | 98 (99) | 100 (100) |
| *R*_merge_ (%)^b^ | 7.4 (66.9) | 18.7 (44.8) |
| I/σ (I) | 16.81 (5.93) | 12.33 (6.91) |
| **Refinement statistics** |  |  |
| Resolutions (Å) | 34.82-1.9 (1.96-1.9) | 47.65-2.24 (2.25-2.18) |
| Reflection used in refinement | 33170 (3315) | 19741 (1926) |
| *R*_work_ (%)^c^ | 16.35(15.45) | 17.18 (22.28) |
| *R*_free_ (%)^d^ | 19.47 (17.98) | 23.71(30.08) |
| Number of non-hydrogen atoms | 3359 | 3280 |
| Number of imidazoles | 2 | 0 |
| Number of acetates | 3 | 2 |
| Number of water | 361 | 357 |
| Number of protein residues | 388 | 378 |
| **RMSD** |  |  |
| Bond lengths (Å) | 0.014 | 0.009 |
| Bond angles (°) | 1.34 | 1.00 |
| Average B-factor (Å^2^) | 27.30 | 21.92 |
| Ramachandran favored (%) | 97.92 | 97.86 |
| Ramachandran allowed (%) | 2.08 | 2.14 |
| Ramachandran outliers (%) | 0 | 0 |
| PDB code | 7C23 | 7C29 |

1. AlinE4

| Items | AlinE4 | AlinE4-D162A | AlinE4-S13A |
| --- | --- | --- | --- |
| **Data collection** |  |  |  |
| Wavelength | 0.97931 | 1.06881 | 0.97915 |
| Resolutions (Å) | 48.46-1.18 (1.21-1.18)^a^ | 48.42-1.55 (1.61-1.55) | 40.00-1.75 (1.84-1.75) |
| Space group | P 4_2_ 2_1_ 2 | P 4_2_ 2_1_ 2 | P 4_2_ 2_1_ 2 |
| Unit cell (Å, °) | a=79.783, b=79.783, c=61.010  α=β=γ=90 | a=79.810 b=79.810, c=60.920  α=β=γ=90 | a=79.810, b=79.810, c=60.820  α=β=γ=90 |
| Unique reflections | 64670 (4370) | 29082 (4174) | 20419 (2929) |
| Completeness (%) | 99.2 (92.7) | 100 (100) | 100 (100) |
| *R*_merge_ (%)^b^ | 7.3 (64.5) | 9.6 (26.2) | 21.6 (76.7) |
| I/σ (I) | 20.0 (3.2) | 32.71 (12.73) | 12.9 (7.6) |
| **Refinement statistics** |  |  |  |
| Resolutions (Å) | 48.46-1.18 (1.22-1.18) | 48.42-1.55 (1.61-1.55) | 39.90-1.75 (1.81-1.75) |
| Reflection used in the refinement | 64487 (5992) | 29037 (2859) | 20382 (1988) |
| *R*_work_ (%)^c^ | 14.11(22.02) | 13.19 (9.62) | 14.14(13.74) |
| *R*_free_ (%)^d^ | 16.29 (23.79) | 16.84(14.68) | 19.47 (20.69) |
| Number of non-hydrogen atoms | 1787 | 1721 | 1660 |
| Number of Cd^2+^ | 3 | 4 | 6 |
| Number of acetates | 4 | 2 | 1 |
| Number of glycerol | 0 | 1 | 3 |
| Number of water | 221 | 188 | 151 |
| Number of protein residues | 189 | 189 | 189 |
| **RMSD** |  |  |  |
| Bond lengths (Å) | 0.018 | 0.016 | 0.016 |
| Bond angles (°) | 2.00 | 1.93 | 1.83 |
| Average B-factor (Å^2^) | 16.30 | 16.30 | 15.96 |
| Ramachandran favored (%) | 98.93 | 98.93 | 98.40 |
| Ramachandran allowed (%) | 1.07 | 1.07 | 1.60 |
| Ramachandran outliers (%) | 0 | 0 | 0 |
| PDB code | 7C82 | 7C84 | 7C85 |

^a^ Statistics for the highest-resolution shell are shown in parentheses.

^b^ *R*_merge_=Σ |Ii − < I> |/Σ |I|, where I_i_ is the intensity of an individual reflection and I is the average

^c^ *R*_work_=Σ ||F_o_| − |F_c_||/Σ |F_o_|, where F_o_ and F_c_ are the observed and calculated structure factors for reflections, respectively.

^d^ *R*_free_ was calculated as *R*_work_ using the 5% of reflections that were selected randomly and omitted from refinement.

**Table S2. The metal ion concentration in AlinE4 crystal measured by the electrochemistry analysis method.**

| Sample ID | Ion | Reported concentration (mg/L) |
| --- | --- | --- |
| 1 | Cd^2+^ | 14.3 |
|  | Ni^2+^ | 0.01 |
| 2 | Cd^2+^ | 18.18 |
|  | Ni^2+^ | 0.007 |

**
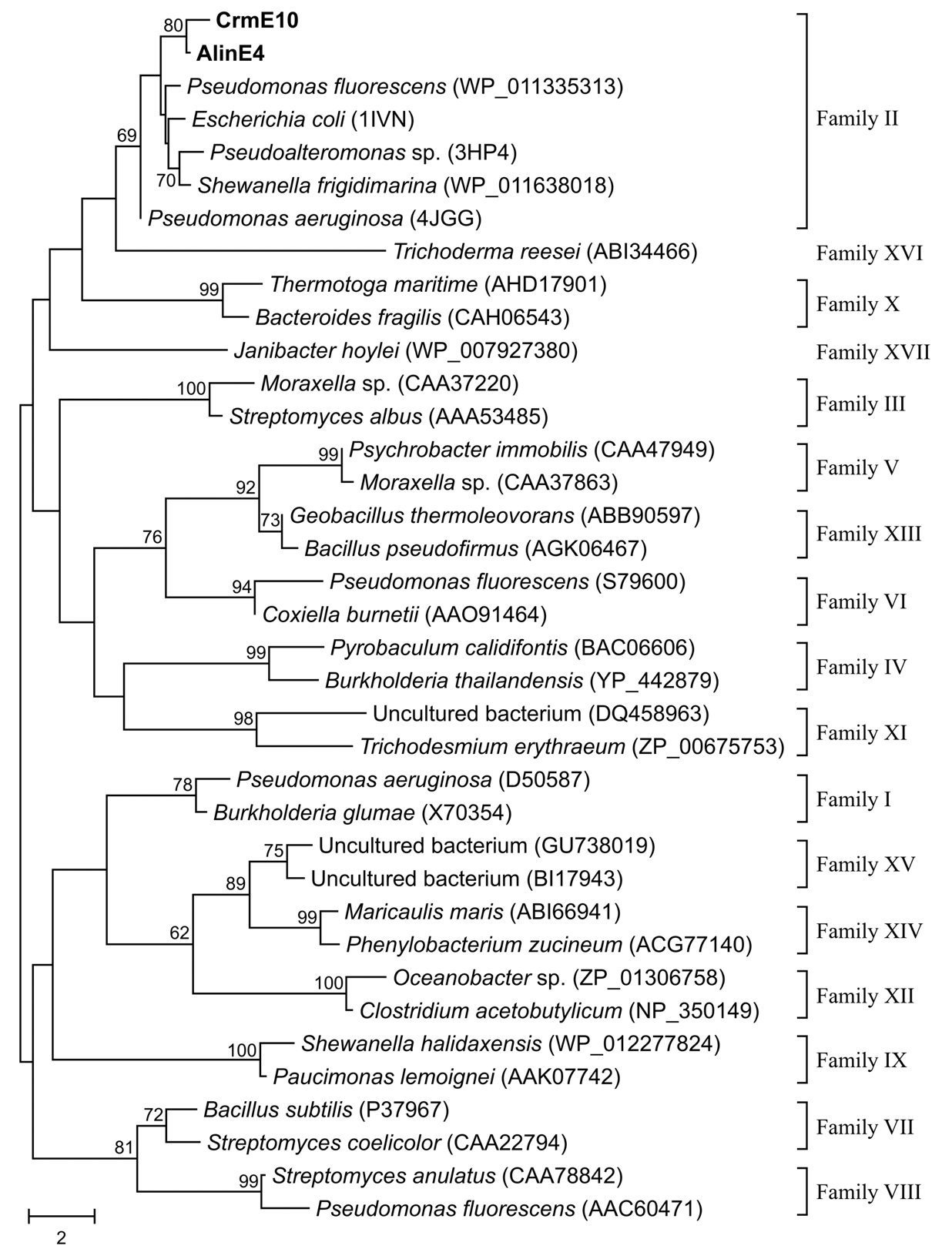
**

**Fig. S1 Neighbor-joining phylogenetic tree of CrmE10 and AlinE4**. The tree was constructed using MEGA software. Bootstrap values are based on 1000 replicates, and only values >50% are shown. The scale bar indicates the number of amino acid substitutions per site.


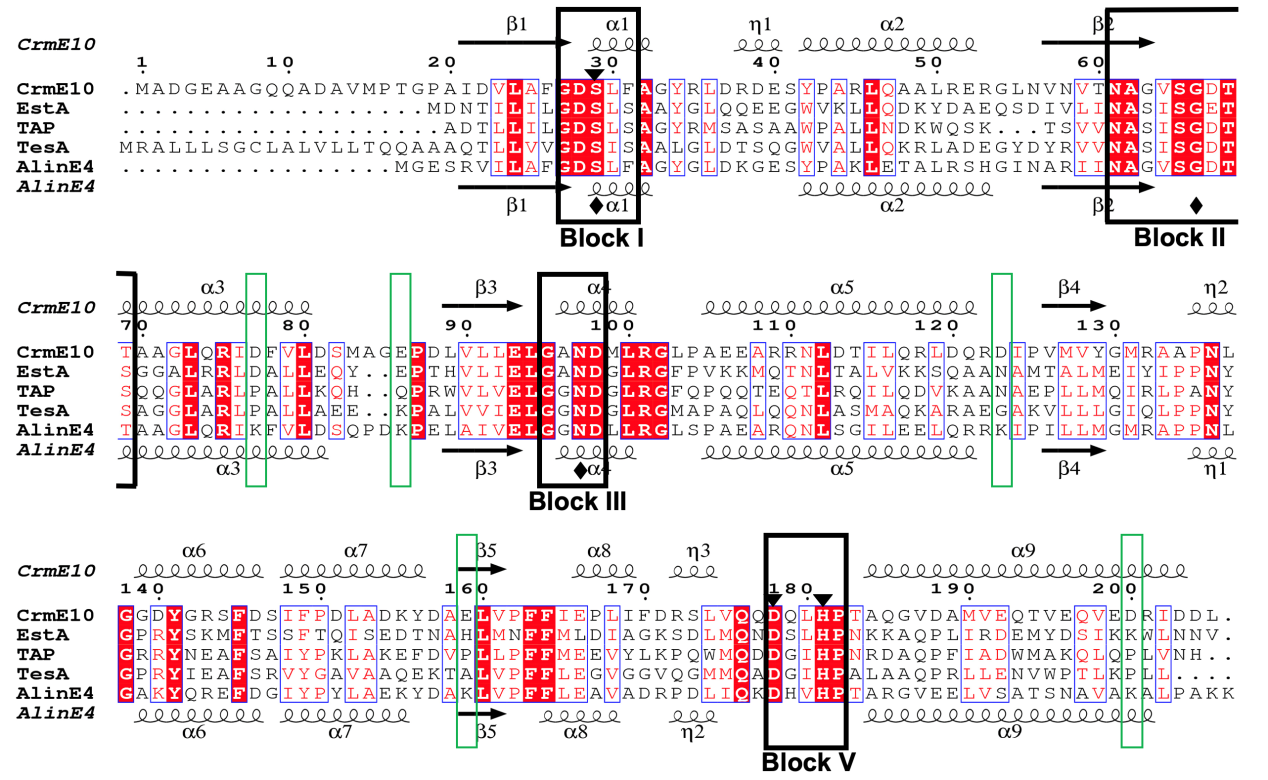


**Fig. S2 Structure-based sequences alignment of CrmE10, AlinE4, and other esterases.** The enzymes are shown below: EstA (PDB code: 3HP4, from *Pseudoalteromonas* sp.), TAP (PDB code: 1IVN, from *Escherichia coli*), and TesA (PDB code: 4JGG, from *Pseudomonas aeruginosa*). Sequence alignment was performed using the ClustalX and ESpript v.3.0 programs. Identical and similar residues are shown in white text on a red background and in red text on a white background, respectively. The secondary structure elements, including α-helix, β-strand, and 3_10_-helix are denoted as α, β, and η, respectively. Consensus blocks, I, II, III and V, related to the catalytic reaction, are marked by black boxes. Five key residues related to the catalytic activity are marked by a green box. The triangles and diamonds indicate the residues in the catalytic triad (Ser-Asp-His) and oxyanion hole (Ser-Gly-Asn), respectively.


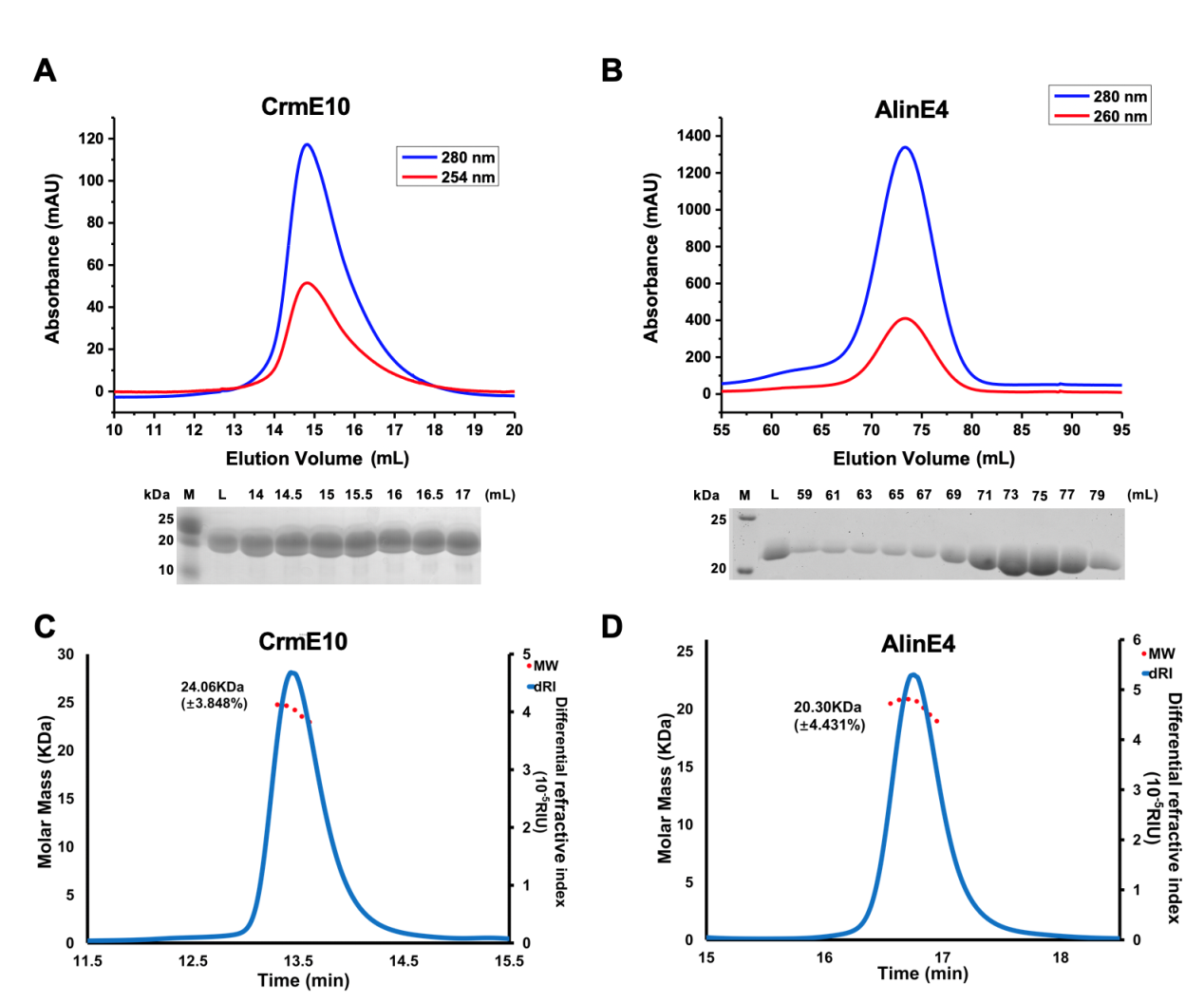


**Fig. S3 Gel-filtration and MALS profiles of CrmE10 and AlinE4.** (A) and (B) are gel filtration profiles (Top) and SDS-PAGE (Bottom) of CrmE10 and AlinE4, respectively. (C) and (D) are MALS results of CrmE10 and AlinE4, respectively. The calculated molecular weight by MALS showed both CrmE10 and AlinE4 are monomer in solution.


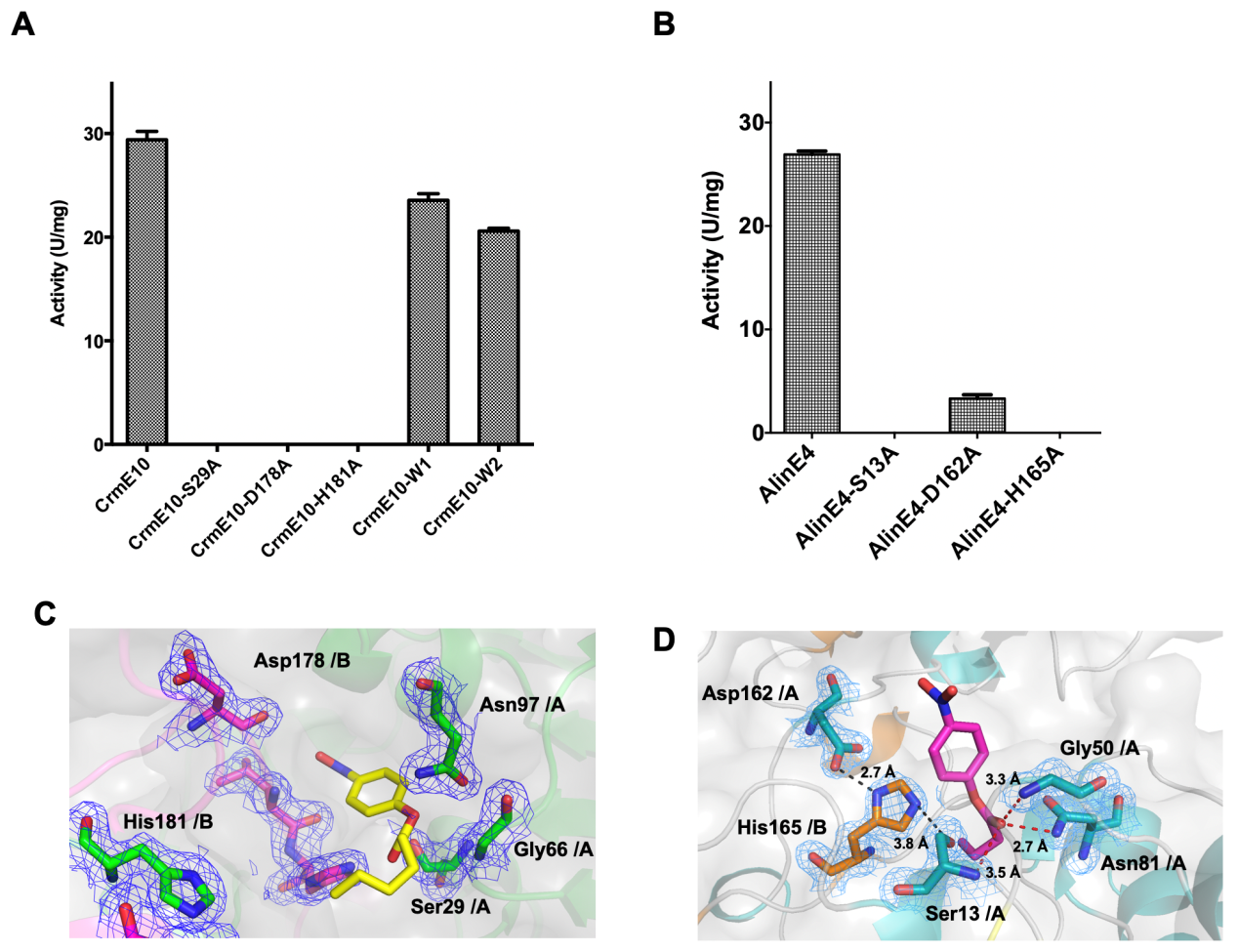


**Fig. S4 Dimerization contributes to the enzymatic activities of CrmE10 (PDB: 7C23) and AlinE4 (PDB: 7C82).** (A) Enzymatic activity of WT CrmE10 and its mutants. (B) Enzymatic activity of WT AlinE4 and its mutants. (C) The structural model of CrmE10 and its auto-docked substrate *p*-NP hexanoate (yellow sticks). (D) The structural model of AlinE4 and its auto-docked substrate *p*-NP butyrate (magenta sticks). Residue His165 of chain B forms hydrogen bonds with Ser13 and Asp162 of chain A (black dotted lines). The substrate molecule forms hydrogen bonds with Gly50 and Asn81 of chain A (red dotted lines). The electronic map is contoured to 1.0 σ at the 2*Fo-Fc* map.


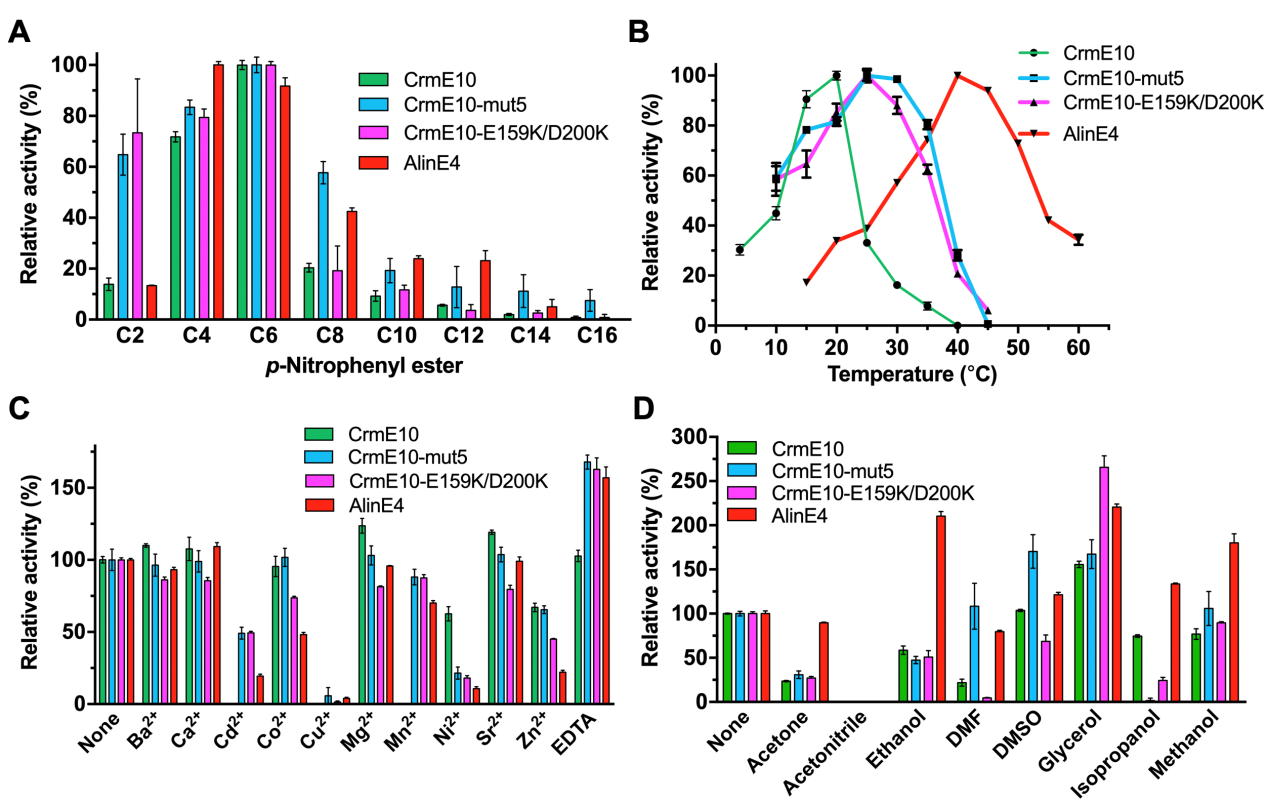


**Fig. S5 Enzymatic Characterizations of Structure-based CrmE10 mutants.** (A) Enzymatic activities toward substrates with various chain lengths of *p*-nitrophenyl (*p-*NP) esters. The value toward *p-*NP hexanoate (CrmE10 and its mutants) and *p-*NP butyrate (AlinE4) were taken as 100%. (B) Effects of temperature on enzyme activities. Enzymatic activity was determined with a series of temperatures. The values obtained at 20 °C (CrmE10), 25 °C (CrmE10 mutants) and 40 °C (AlinE4) were taken as 100%. (C) Effects of different metal ions on the activities. The values obtained without ions in the reaction mixture were taken as 100%. (D) Effects of organic solvents on the activities. The values obtained without organic solvent were taken as 100%.


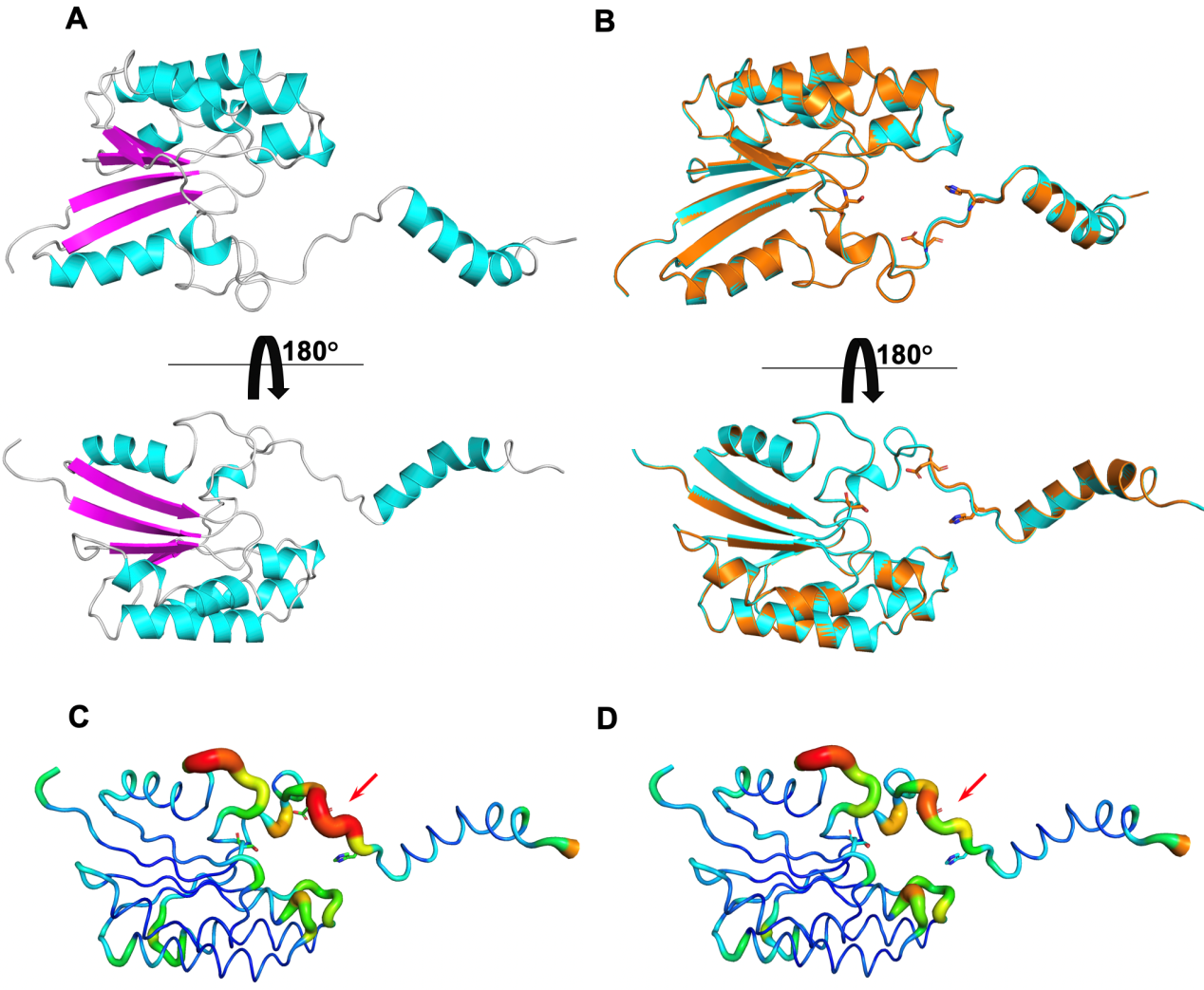
.

**Fig. S6 Structural comparison of AlinE4 (PDB: 7C82) and its mutant AlinE4-D162A (PDB: 7C84).** (A) Cartoon representation of AlinE4-D162A. The α-helices and β-strands of AlinE4-D162A are depicted in cyan and magenta, respectively. (B) The structural superposition of AlinE4 (green) and AlinE4-D162A (cyan). The B-factor distributions of AlinE4 and AlinE4-D162A are shown in (C) and (D) with the tubing model. Wider and redder tubing corresponds to higher B-factor. Red arrows indicate the flexible loops between 3_10_-helix η3 and α-helix α9 of AlinE4.
